# Investigating the Role of Upward Comparisons and Self-compassion on Stigma in People With Acne: Cross-sectional Study

**DOI:** 10.2196/45368

**Published:** 2023-04-12

**Authors:** Kate Adkins, Paul G Overton, Julia Moses, Andrew Thompson

**Affiliations:** 1 Department of Psychology University of Sheffield Sheffield United Kingdom; 2 Department of History University of Sheffield Sheffield United Kingdom

**Keywords:** acne, stigma, appearance comparisons, self-compassion, social media, psychological distress, stigmatization, acne symptoms, symptoms, Facebook, Instagram, skin, engagement, appearance, distress

## Abstract

**Background:**

The use of image-laden social media is hypothesized as being implicated in psychological distress in individuals with conditions affecting their appearance. However, relatively little is known about the mechanisms involved in this relationship.

**Objective:**

This cross-sectional study examined the relationship between photo-orientated social media use and feelings of stigmatization in adults with acne, and tested whether upward skin comparisons mediate and self-compassion moderates this relationship.

**Methods:**

Adults (N=650) with acne symptoms completed web-based measures of social media use (daily Facebook or Instagram use, Facebook function use), self-compassion, skin appearance comparisons, and internalized stigmatization.

**Results:**

Moderated-mediation and mediation analyses indicated that there was a significant indirect effect of Facebook photo use on stigmatization, mediated by upward appearance comparisons (estimation of indirect effect 11.03, SE 5.11, 95% CI 1.19-21.12). There was no significant relationship between Instagram use and feelings of stigmatization (estimation of indirect effect 0.0002, SE 0.005, 95% CI −0.011 to 0.009), yet upward appearance comparisons predicted feelings of stigmatization (*B*=0.99, *P*<.001). Self-compassion did not moderate the indirect or direct relationships between photo-orientated social media use and stigma. However, self-compassion was negatively correlated with upward appearance comparisons and feelings of stigmatization in both Facebook and Instagram users.

**Conclusions:**

The way that individuals engage with social media, and in particular make appearance comparisons, should be considered when working with individuals with skin-related distress. Interventions aimed at boosting self-compassion and reducing appearance comparisons may provide avenues for protecting against feelings of stigma.

## Introduction

Individuals living with visible skin conditions, including acne vulgaris (acne), can experience stigmatization from others (enacted stigma) and internalize feelings of stigmatization (felt stigma) [1-9]. Individual accounts of stigma [2,4,5] are corroborated by experimental research that indicates an implicit preference for clear skin and negative assumptions about individuals with acne [6-8]. Stigma has important implications for psychosocial well-being; surveys of individuals with acne report that felt stigma explains the largest variance (25%-36%), beyond other predictors (eg, perceived severity and gender), across quality-of-life domains: self-perception, social, and emotional [1,9]. Similar findings have been reported in populations with other skin conditions [10,11].

The psychological burden of acne has been well documented [12]. Of the various domains of impact, the impact of acne on self-perception has been the most widely reported. So, acne leads to appearance-related distress [13], body image disturbance [14], and feeling unattractive or ugly [15]. In order to reduce the psychological burden associated with acne, it is essential to understand the psychological mechanisms involved in acne stigmatization. Understanding these mechanisms using psychological theory has the potential to provide theoretical “proof of concept” for suitable targets for psychological therapy. Although dermatological (severity and duration) [1,16,17] and demographic (employment status, age, relationship status, and gender) [1,16-20] variables have limited predictive power, wider sociocultural factors are likely to play a more significant role. Sociocultural factors, including contemporary media, are theorized to influence societal norms and appearance ideals, contributing to the stigmatization of individuals who are unable to meet these ideals [21-23]. Correspondingly, within a qualitative study, participants with acne, eczema, and psoriasis described a pervasive media ideal of perfect skin [24]. Failure to meet this ideal was related to greater depression and stigmatization in female, but not male participants [24]. However, there was no distinction between media platforms or investigation of the specific psychological mechanisms that might be involved.

Web-based activity now plays a major role in our lives. As of 2021, a total of 88% of all UK adults possessed a social media account [25]. Facebook remains the most popular site (66% of adult social media users report using Facebook) [25]. Instagram, an image-based platform allowing users to digitally manipulate and share images, is growing in popularity (48% of adult social media users) [25]. Acne frequently affects adolescents [26], a group who are particularly engaged with social media [27].

A number of studies have established a relationship between Facebook use and psychosocial outcomes [28], with the role of individual difference variables in social media use and behavior showing greater promise in explaining the impact of such media, over and above simple usage [29-31]. For example, higher photo-function use, over and above total Facebook usage, has been reported to predict greater weight dissatisfaction, thin-ideal internalization, appearance comparison, and self-objectification [30]. Similar findings are emerging for Instagram use, with undergraduate students experimentally exposed to idealized Instagram images of celebrities and peers, as opposed to Instagram travel pictures, reporting increased body dissatisfaction and negative mood, mediated by appearance comparisons [32].

Early theories of social comparison posited that humans have an innate drive to compare themselves with others as part of maintaining group relationships [33]. As such, a perceived sense of difference may act as a threat, which may drive unhelpful comparisons. Social comparison theory has been expanded to include appearance-based comparisons, and downward and upward comparisons, where individuals compare themselves with others they perceive as superior (upward) or inferior (downward) [34]. Social and upward appearance comparisons have been established as predictors of body dissatisfaction [35], body-shaming [36], and mediators between media exposure to idealized images and body dissatisfaction [30,31,37-39]. Social comparisons are reported to be an important mechanism in the way individuals with a stigmatized identity evaluate themselves [40] and have been theorized as a core process implicated in skin-shaming [41]. Further, Kellett and Gibert [41] have anecdotally found that patients they treat who are distressed in relation to the appearance of their skin condition are often engaging in making such comparisons. However, the relationship between skin-specific appearance comparisons, social media use, and felt stigmatization has thus far not been investigated, nor has the related role of protective factors like self-compassion.

Self-compassion is theorized to involve 3 main components that influence how we treat ourselves and react to difficulties: self-kindness, mindfulness, and common humanity [42]. Self-compassion may act as a protective factor against psychosocial distress in stigmatized populations [43,44], and intervention-based studies using compassion-based training have shown promise in reducing feelings of shame in participants with acne [45].

As a consequence of the lack of research on social media and acne stigmatization, we conducted a web-based survey to investigate the relationship between photo-related social media use and felt stigma in people with acne. We hypothesized that relative photo-based social media use (Facebook photo activity and total time on Instagram), not total time on Facebook, would be related to felt stigmatization: (1) individuals who spend proportionally more of their time using photo- or appearance-orientated social media will have higher levels of felt stigmatization, (2) this relationship will be mediated by upward skin appearance comparisons, and (3) these relationships will be moderated by self-compassion.

## Methods

### Ethical Approval

Ethical approval for this cross-sectional study was granted by the University of Sheffield ethics committee (reference 011937).

### Sample and Recruitment

Participants with acne symptoms were recruited between February and March 2017 from a convenience community sample and offered entry into a prize draw. The study was advertised across multiple social media platforms, UK skin charities, web-based recruitment platforms, university volunteer lists, and an undergraduate credit system. To be included in the study, participants were required to meet the following inclusion criteria: (1) 16 years or older, (2) current symptoms of acne, (3) living in the United Kingdom or have UK citizenship, and (4) know sufficient English to complete the survey.

A power analysis for multiple regression with 10 predictors indicated that at least 253 participants would be needed to achieve 80% power with a significance level of .05 to detect a small effect size, *r*=0.25.

### Procedure

Participants completed counterbalanced self-report measures of demographics, acne history, Facebook use, Facebook function use, Instagram use, skin-related upward or downward comparisons, self-compassion, and acne stigma via a web-based survey using Qualtrics.

### Measures

#### Demographics and Acne History

Participants provided information about their gender, age, ethnicity, educational level and relationship status, and their acne history, including perceived severity, location of symptoms (categorized as visible and nonvisible), and whether they had received a formal diagnosis or acne treatment from a health professional. Perceived severity was measured using a question based on the fifth question of the Cardiff Acne Disability Index [46], which includes a question about the degree to which acne is a problem for the participant.

#### Facebook Use

Participants were asked whether they had used Facebook within the past month. If participants answered “yes,” they were asked to estimate the amount of time they spent on Facebook in the past week. Daily Facebook use was calculated using the following formula: Average daily Facebook use = (Number of days Facebook used × Time spent on Facebook on these days) / 7.

#### Relative Facebook Photo Activity

The Facebook Questionnaire functions [30] assessed relative levels of photo activity compared with nonphoto activities on Facebook. The scale consists of 24 items (α=.86), scored on a 6-point Likert scale. The appearance- or photo-activity subscale is formed of 8 items (α=.76) related to appearance-specific photo activity. Proportionate Facebook photo activity was calculated by dividing the total for the photo-activity subscale by the total for all items. Scores range from 0 to 1; scores closer to 1 indicate a higher proportion of time spent using photo-related functions on Facebook.

#### Instagram Use

Instagram is an image-based platform. Instagram photo activity was measured using the average time spent on Instagram per day. Participants were asked whether they had used Instagram in the past month. If participants answered “yes,” they were asked to estimate the amount of time they spend on Instagram. Daily Instagram use was calculated using the following formula: Average daily Instagram use = (Number of days Instagram used × Time spent on Instagram on these days) / 7.

#### Skin-Based Comparisons

The Upward and Downward Appearance Comparison Scales (UPACS, DACS) [34] measure both upward and downward appearance-based comparisons in relation to shape and size. The UPACS and DACS were adapted to measure skin comparisons, and one social media question was added each to the UPACS (“On social media I tend to compare how my skin looks to photographs of people with clearer skin than me”) and the DACS (“On social media I tend to compare how my skin looks to photographs of people with worse skin than me”). Both items correlated highly with the other items in the scales and did not reduce reliability. The adapted UPACS and DACS each contained 9 items (UPACS: α=.93; DACS: α=.94), scored on a 5-point Likert scale. Higher scores indicated higher levels of upward appearance comparisons and higher levels of downward appearance comparisons.

#### Self-compassion

Self-compassion was measured using the Self-Compassion Scale Short Form [47]. The scale comprises 3 domains: self-kindness versus self-judgment, common humanity versus isolation, and mindfulness versus overidentification. The items (α=.86) are scored on a 5-point Likert scale. Higher scores indicate greater self-compassion.

#### Skin-Related Stigma

Stigma was measured using the total score on the Feelings of Stigmatization Questionnaire [13], originally developed to assess felt stigmatization in patients with psoriasis. The scale has been adapted and previously used in the context of acne [1]. Amendments to the scale for this study involved replacing the term “psoriasis” with “acne” and replacing the term “patient” with “person” as the survey uses a community sample. One question unrelated to acne (“I do not mind when a family member gives me a vacuum cleaner to clean up the scales that fall from my psoriatic skin”) was deleted. The adapted measure contained 32 questions (α=.92), scored on a 6-point Likert scale. Higher scores indicate greater felt stigmatization.

### Analytic Strategy

Data were analyzed using SPSS version 23 (IBM Corp).

Descriptive statistics were calculated using percentages for categorical variables and means and SDs for continuous variables. Demographic and acne history variables were assessed for covariance with felt stigma using *t* tests, ANOVAs, and bivariate correlations as appropriate. Relationships between the predictor variables, the mediator variables, and the outcome variables were initially tested using bivariate correlations. Nonparametric tests were used when analyzing average Facebook and Instagram use, as normality tests indicated that they were nonnormally distributed. Significant covariates were entered into subsequent analyses.

**Figure 1:**
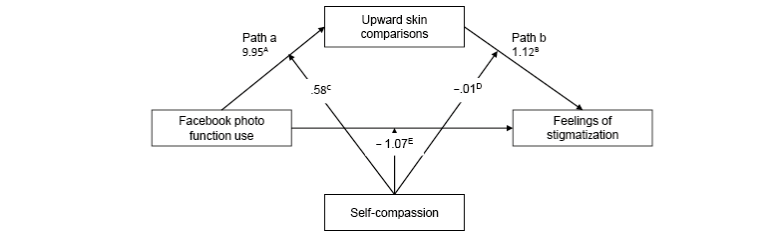
Moderated-mediation model for Facebook photo use on acne stigma via upward appearance comparison, with self-compassion as the moderator for each path (N=591). The numbers presented in the figure represent unstandardized β values, as recommended by Hayes [48]. The numbers on the arrows intercepting paths a, b, and c represent the unstandardized β values for the interaction effects. (A) *P*=.03, (B) *P*<.001, (C) *P*=.20, (D) *P*=.25, (E) *P*=.42. For clarity, covariates are not included in the figure. The covariates that were controlled for on each pathway were gender, severity, acne diagnosis, and downward skin comparison.

**Figure 2:**
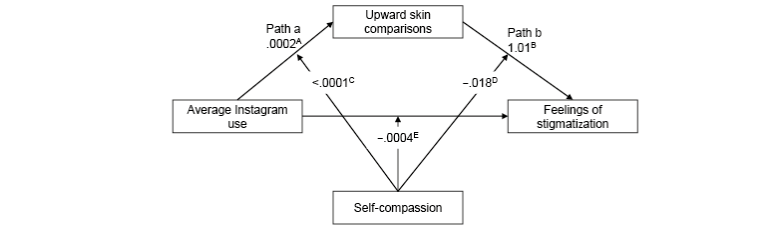
Moderated-mediation model for average Instagram use on acne stigma via upward appearance comparison, with self-compassion as the moderator for each path (N=429). The numbers presented in the figure represent unstandardized β values, as recommended by Hayes [48]. The numbers on the arrows intercepting paths a, b, and c represent the unstandardized β values for the interaction effects. (A) *P*=.98, (B) *P*<.001, (C) *P*=.98, (D) *P*=.25, (E) *P*=.83. For clarity, covariates are not included in the figure. The covariates that were controlled for on each pathway were gender, severity, acne diagnosis, and downward skin comparison.

**Figure 3:**
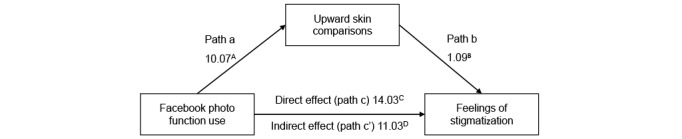
Mediation model for Facebook photo use on acne stigma via upward appearance comparison (N=591). The numbers presented in the figure represent unstandardized β values, as recommended by Hayes [48]. (A) *P*=.03, (B) *P*<.001, (C) *P*=.29, (D) 95% CI –11.86 to 39.93. For clarity, covariates are not included in the figure. The covariates that were controlled for on each pathway were gender, severity, acne diagnosis, downward skin comparison, and self-compassion.

**Figure 4:**
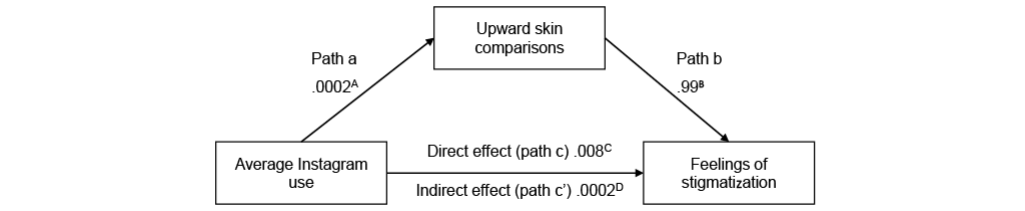
Mediation model for average Instagram use on acne stigma via upward appearance comparison (n=429). The numbers presented in the figure represent unstandardized β values, as recommended by Hayes [48]. (A) *P*=.98, (B) *P*<.001, (C) *P*=.53, (D) 95% CI –0.011 to 0.009. For clarity, covariates are not included in the figure. The covariates that were controlled for on each pathway were gender, severity, acne diagnosis, downward skin comparison, and self-compassion.

Hypothesized relationships between photo-related social media activity, upward appearance comparisons, self-compassion, and stigmatization were tested using (1) moderated-mediation (Figures 1 and 2) and (2) mediation-only analysis (Figures 3 and 4), using ordinary least-squares path analysis. Analyses were conducted using the PROCESS macro version 3.442 with 10,000 bootstrap samples.

## Results

### Demographics

Of 818 participants who started the survey, 652 participants completed the survey. Two participants were excluded because of unfeasible social media use (≥24 hours per day), so overall, 650 participants (aged 16-56 years; 82.9% female) were included in the analyses (69.7% completion rate). Information on participant demographics and acne history is presented in Tables 1 and 2.

In all, 591 participants reported using Facebook, whereas 428 reported using Instagram. The 2 groups were similar in age (Facebook: mean 24.2, SD 6.4 years; Instagram: mean 23.2, SD 5.7 years). Also, there was no association between the 2 platforms (Facebook and Instagram) in terms of the frequency of participants being a student or not a student (*χ*^2^_1_=0.21, *P*=.65), or White or not White (*χ*^2^_1_=0.82, *P*=.37). However, there were a greater proportion of females in the Instagram group (female vs male + other, *χ*^2^_1_=5.11, *P*=.04) and a higher proportion of people 25 years or older (considered to have adult acne [49]) in the Facebook group (*χ*^2^_1_=5.01, *P*=.03).

Participants with a formal acne diagnosis reported higher stigma (mean 76.43, SD 25.22) compared with participants reporting acne symptoms but without a formal diagnosis (mean 67.91, SD 25.73; *t*_648_=3.88; *P*<.001; Cohen *d*=0.33). Furthermore, Spearman correlations showed a small correlation between self-rated severity and stigmatization (*ρ*_648_=0.33, *P*<.001). However, there was no relationship between the duration of acne symptoms and stigmatization (*ρ*_648_=0.06, *P*=.11).

Free-text responses to the question “Please list any other diagnosed physical or mental health conditions” were coded into yes (1) or no (0) responses on three variables: (1) skin condition, (2) long-term health condition (excluding skin conditions), and (3) mental health conditions. There was no significant effect of other skin conditions on stigmatization (*t*_632_=−0.03, *P*=.98, *d*=0.003). However, respondents disclosing a comorbid long-term health condition also reported significantly greater acne stigma (mean 78.57, SD 24.04) than participants without a long-term health condition (mean 73.03, SD 25.79; *t*_632_=2.15; *P*=.03; *d*=0.22). Furthermore, participants disclosing at least 1 diagnosed mental health condition reported significantly higher levels of stigmatization (mean 84.67, SD 25.29) than participants without (mean 71.37, SD 24.92; *t*_632_=5.4; *P*<.001; *d*=0.53).

Female participants reported higher stigmatization levels (mean 76.17, SD 24.74) compared with male participants (mean 63.34, SD 27.35; *t*_647_=0.49; *P*<.001; *d*=0.49). No other demographic variables were related to stigma, and those that were (gender, acne diagnosis, acne severity, and long-term health condition) were controlled for in moderated-mediation and mediation analyses. Mental health diagnoses were not included as a covariate as higher levels of mental health problems have previously been identified as a consequence of internalized stigmatization in individuals with skin diseases [10,50].

**Table 1:** Participant demographics (N=650).

Demographics and participant characteristics			Participants
**Age (years), mean (SD; range)**			24.47 (6.64; 16-56)
	≥25, n (%)	225 (34.6)	
**Gender, n (%)**			
	Female	539 (82.9)	
	Male	110 (16.9)	
	Other	1 (0.2)	
**Ethnicity, n (%)**			
	White or Caucasian	510 (78.5)	
	Asian	92 (14.4)	
	Mixed	26 (4.0)	
	Black	10 (1.5)	
	Arab	5 (0.8)	
	Latin American	4 (0.8)	
	“Prefer not to answer”	3 (0.5)	
**Employment, n (%)**			
	Student	409 (62.9)	
	Employed	209 (32.2)	
	Both employed and student	5 (0.8)	
	Unemployed or unable to work	12 (1.8)	
	Homemakers or carers	11 (1.7)	
	“Prefer not to answer”	4 (0.6)	
**Education level, n (%)**			
	Undergraduate	246 (37.8)	
	A level or equivalent	206 (31.7)	
	Postgraduate	145 (22.3)	
	GCSE^a^ or equivalent	21 (3.2)	
	Vocational	22 (3.4)	
	Other, unsure, or “prefer not to answer”	10 (1.5)	
**Marital status, n (%)**			
	Single	285 (43.8)	
	In a relationship	196 (30.2)	
	Cohabiting with partner	84 (12.9)	
	Married or civil partnership	79 (12.2)	
	“Other” or “prefer not to answer”	6 (0.9)	

^a^GCSE: General Certificate of Secondary Education.

**Table 2:** Participant acne and health history (N=650).

Acne history and participant characteristics			Participants
Acne duration (months), mean (SD; range)			115 (82.38; 1-480)
**Acne diagnosis, n (%)**			
	Yes	463 (71.2)	
	No or unsure	187 (28.8)	
**Current treatment, n (%)**			
	GP^a^	189 (29.1)	
	Dermatologist	57 (8.8)	
	Gynecologist	4 (0.6)	
	Other health professional	3 (0.5)	
	None	395 (60.7)	
	Prefer not to answer	2 (0.3)	
**Location^b^, n (%)**			
	Visible	638 (98.2)	
	Nonvisible	12 (1.8)	
Subjective severity, mean (SD)^c^			2.31 (0.58)
**Other diagnoses, n (%)**			
	Yes	277 (42.6)	
	No	357 (54.9)	
	“Prefer not to answer”	16 (2.5)	
	Other skin condition(s)	71 (10.9)	
	Long-term health condition(s)	121 (18.6)	
	Mental health condition(s)	129 (19.8)	

^a^GP: general practitioner.

^b^Characterized as visible if the location of acne included their face, scalp, neck, hands, or arm.

^c^Range 1 (not a problem) to 4 (the worst it could be).

### Relationships Between Social Media Use, Appearance Comparisons, Self-Compassion, and Stigma

Table 3 provides bivariate correlations for each outcome variable, time spent on Facebook and Instagram, and relative Facebook photo function use.

As predicted, within Facebook users, photo-related Facebook activity positively correlated with upward appearance comparison and stigmatization, whereas average daily Facebook use was not correlated with Facebook photo activity nor stigmatization. Among Instagram users, average time on Instagram correlated positively with upward appearance comparisons but not stigmatization.

Furthermore, among all respondents, there was a large positive correlation between upward comparisons and stigmatization (*r*_648_=0.53, *P*<.001). Self-compassion was negatively correlated with upward comparisons (*r*_648_=−0.41, *P*<.001), stigmatization (*r*_648_=−0.46, *P*<.001), and Facebook photo activity (*r*_590_=−0.11, *P*=.009), but not average Facebook use (*ρ*_590_=−0.048, *P*=.24) nor Instagram use (*ρ*_427_=0.093, *P*=.06).

Downward comparisons had a small significant correlation with Instagram use, upward comparisons, compassion, and stigma, and were therefore included as a covariate within the models below.

**Table 3:** Bivariate correlations between each of the predictor and outcome variables (N=650).

		1, *ρ*	2, *r*	3, *ρ*	4, *r*	5, *r*	6, *r*	7, *r*
**1. FB use^a^**								
	Coefficient	—^b^						
	*P* value	—						
**2. FB photo^a^**								
	Coefficient	−0.003	—					
	*P* value	.94	—					
**3. Instagram use^c^**								
	Coefficient	0.32^d^	0.16^d^	—				
	*P* value	<.001	.001	—				
**4. UPACS^e^**								
	Coefficient	0.054	0.17	0.12	—			
	*P* value	.19	<.001	.01	—			
**5. DACS^f^**								
	Coefficient	0.062	0.051	0.12	0.38	—		
	*P* value	.13	.22	.01	<.001	—		
**6. Compassion**								
	Coefficient	−0.048	−0.11	−0.093	−0.41	−0.18	—	
	*P* value	.24	.009	.06	<.001	<.001	—	
**7. Stigma**								
	Coefficient	0.06	0.14	0.068	0.53	0.295	−0.46	—
	*P* value	.230	.001	.162	<.001	<.001	<.001	—
Values		Median 30 (IQR 7.5-52.5; range 0-700)	Mean 0.39 (SD 0.069; range 0.00-0.79)	Median 30 (IQR 5.0-55.0; range 0-600)	Mean 33.49 (SD 8.38; range 9-45)	Mean 24.61 (SD 9.01; range 9-45)	Mean 32.55 (SD 7.64; range 12-57)	Mean 73.98 (SD 25.64; range 7-145)

^a^Excluding participants who reported not using Facebook (n=592). FB use: average Facebook use per day; FB photo: Facebook Questionnaire functions.

^b^Not available.

^c^Excluding participants who reported not using Instagram (n=429). Instagram use: average Instagram use per day.

^d^Excluding participants who did not use both Facebook and Instagram (n=403)

^e^UPACS: Upward Appearance Comparison Scale.

^f^DACS: Downward Appearance Comparison Scale.

### Mediation and Moderated-Mediation Analyses

Moderated-mediation analyses were conducted to assess the conditional direct and indirect effects of photo-related social media activity on stigmatization at values of self-compassion 1 SD below the mean, the mean, and 1 SD above the mean.

The results of the moderated-mediation analysis (Figure 1, Table 4) did not support a model of moderated mediation for Facebook photo activity and acne stigma. Interactions of self-compassion on path a (*B*=0.58, *P*=.20), path b (*B*=−0.01, *P*=.25), or path c (*B=*−1.07, *P=*.42) were nonsignificant. Likewise, the results of the moderated-mediation analysis (Figure 2, Table 5) did not support a model of moderated mediation for Instagram use and acne stigma. Interactions of self-compassion on path a (*B*<0.001, *P*=.98), path b (*B*=−0.018, *P*=.25), or path c (*B*=0.0004, *P*=.83) were nonsignificant.

Subsequently, simpler mediation models were explored. Conditional direct and indirect effects of photo-related social media activity on stigmatization were assessed with self-compassion as a covariate. Mediation analysis (Figure 3; Table 6) indicated that there was a significant indirect effect of Facebook photo use on stigmatization via upward appearance comparison (estimation of indirect effect 11.03, SE 5.11, 95% CI 1.19-21.12). There was no significant direct (estimation of direct effect 14.03, SE 13.19, 95% CI −11.86 to 39.93) or total effect (estimation of total effect 25.06, SE 15.14, 95% CI −4.68 to 54.80) of Facebook photo activity on stigmatization. Furthermore, self-compassion predicted lower levels of upward appearance comparison (*B*=−0.34, *P*<.001) and stigmatization (*B*=−0.85, *P*<.001).

Conversely, mediation analysis (Figure 4; Table 7) indicated that there was no significant direct (estimation of direct effect 0.008, SE 0.013, 95% CI −0.017 to 0.033), total (estimation of total effect 0.008, SE 0.013, 95% CI −0.018 to 0.034), or indirect (estimation of indirect effect 0.0002, SE 0.005, 95% CI −0.011 to 0.009) effect of Instagram use on stigmatization via upward appearance comparison. However, upward appearance comparisons continued to predict stigmatization in Instagram users (*B*=0.99, *P*<.001). Self-compassion also continued to predict lower levels of upward appearance comparison (*B*=−0.33, *P*<.001) and stigmatization (*B*=−1.04, *P*<.001).

**Table 4:** The conditional direct and indirect effects of Facebook photo function use on stigmatization at values of self-compassion 1 SD below the mean, the mean, and 1 SD above the mean (N=591).

Value of self-compassion	Direct effect			Indirect effect	
	*B* (SE)	95% CI	*B* (SE)		95% CI
−7.61	22.83 (18.17)	−12.84 to 58.51	6.62 (6.92)		−8.21 to19.52
0.0000	14.66 (13.60)	−12.05 to 41.37	11.11 (5.15)		1.02 to 21.25
7.61	6.49 (15.79)	−24.52 to 37.49	14.83 (6.21)		2.85 to 27.39

**Table 5:** The conditional direct and indirect effects of Instagram use on stigmatization at values of self-compassion 1 SD below the mean, the mean, and 1 SD above the mean (N=429).

Value of self-compassion	Direct effect		Indirect effect	
	*B* (SE)	95% CI	*B* (SE)	95% CI
−7.35	0.005 (0.022)	−0.038 to 0.048	0.0004 (0.008)	−0.018 to 0.015
0.0000	0.008 (0.01)	−0.019 to 0.035	0.0005 (0.0056)	−0.011 to 0.01
7.35	0.011 (0.016)	−0.021 to 0.043	0.0006 (0.0083)	−0.021 to 0.12

**Table 6:** Summary of the mediation analysis for Facebook photo activity (N=591).

Variable			*B* (SE)		*P* value		95% CI
**Path a: Outcome: UPACS^a^ (*R*^2^=0.33, *P*<.001)**							
	Constant	27.07 (2.85)		<.001		21.47 to 32.67	
	Facebook photo activity	10.07 (4.6)		.03		1.02 to 19.11	
	DACS^b^	0.28 (0.035)		<.001		0.21 to 0.35	
	Severity	1.36 (0.52)		.009		0.34 to 2.39	
	Gender	3.47 (0.91)		<.001		1.68 to 5.26	
	Self-compassion	−0.34 (0.042)		<.001		−0.42 to −0.26	
	Diagnosis	1.42 (0.65)		.03		0.014 to 2.7	
	Long-term health condition	−0.73 (0.79)		.36		−2.28 to 0.82	
**Path b: Outcome: stigma (*R*^2^=0.42, *P*<.001)**							
	Constant	29.37 (8.59)		<.001		12.49 to 46.25	
	UPACS	1.09 (0.12)		<.001		0.87 to 1.32	
	Facebook photo use	14.03 (13.19)		.28		−11.86 to 39.93	
	DACS	0.25 (0.1)		.02		0.046 to 0.45	
	Severity	9.56 (1.54)		<.001		6.53 to 12.59	
	Gender	−1.45 (2.21)		.51		−5.8 to 2.9	
	Self-compassion	−0.85 (0.12)		<.001		−1.08 to −0.61	
	Diagnosis	2.5 (1.85)		.17		−1.11 to 6.15	
	Long-term health condition	3.68 (2.18)		.09		−0.6 to 7.96	

^a^UPACS: Upward Appearance Comparison Scale.

^b^DACS: Downward Appearance Comparison Scale.

**Table 7:** Summary of the mediation analysis for Instagram use (N=429).

Variable			*B* (SE)		*P* value		95% CI
**Path a: Outcome: UPACS^a^ (*R*^2^=0.30, *P*<.001)**							
	Constant	31.27 (2.98)		<.001		25.5 to 37.04	
	Instagram use	0.0002 (0.005)		.98		−0.01 to 0.01	
	DACS^b^	0.25 (0.044)		<.001		0.16 to 0.33	
	Severity	2.01 (0.59)		<.001		0.84 to 3.17	
	Gender	3.02 (1.17)		.10		0.73 to 5.3	
	Self-compassion	−0.33 (0.048)		<.001		−0.43 to –0.24	
	Diagnosis	0.8 (0.75)		.28		−0.73 to 2.27	
	Long-term health condition	−0.88 (0.82)		.29		−2.5 to 0.74	
**Path b: Outcome: stigma (*R*^2^=0.39, *P*<.001)**							
	Constant	49.21 (10.13)		<.001		29.29 to 69.14	
	UPACS	0.99 (0.15)		<.001		0.7 to 1.29	
	Instagram use	0.008 (0.01)		.53		−0.02 to 0.33	
	DACS	0.1 (0.12)		.39		−0.13 to 0.33	
	Severity	8.83 (1.75)		<.001		5.38 to 12.27	
	Gender	−1.42 (3.17)		.65		−7.65 to 4.81	
	Self-compassion	−1.04 (0.16)		<.001		−1.35 to −0.74	
	Diagnosis	4.4 (2.14)		.04		0.2 to 8.6	
	Long-term health condition	0.66 (2.58)		.80		−4.4 to 5.72	

^a^UPACS: Upward Appearance Comparison Scale.

^b^DACS: Downward Appearance Comparison Scale.

## Discussion

This study sought to investigate the relationship between photo-related social media use and feelings of stigmatization in adults with acne. Consistent with the hypothesis, a higher proportion of time engaged in Facebook photo activity, not overall time on Facebook, was correlated with greater feelings of stigmatization in participants with acne, and this relationship was mediated by upward appearance comparison. However, this was the case for Facebook users only, as no such relationship was identified for participants using Instagram; yet among these users, upward appearance comparisons predicted felt stigmatization. Interestingly, although greater self-compassion was related to lower stigmatization, it did not moderate the relationship between social media photo use and acne-related stigma in either Facebook or Instagram users.

It is unclear why there was an indirect relationship between relative Facebook photo activity and stigma but not between Instagram use and stigma. This may reflect the choice of measures, as the measure of Instagram usage did not differentiate between types of usage [32]. Future research should therefore distinguish between types of Instagram use.

Existing research on stigmatization in individuals with skin conditions has primarily focused on stigmatization as a predictor of depression and impaired quality of life, and demographic and condition variables as predictions of stigma. Such research has consistently identified perceived stigmatization as a predictor of reduced quality of life and psychological morbidity. In line with previous research, this study identified associations with stigmatization and gender, perceived severity, and possessing a diagnosis of acne [10,17,20]. However, skin-related comparisons and self-compassion were more consistently associated with felt stigmatization. These findings suggest that the way individuals interact with social media is more important than how long they use it for understanding the associations between social media and well-being. This is important as such meta-cognitive processes are amenable to modification within psychological therapy.

This study has a number of limitations. Clearly, the cross-sectional design prevents comment on causation, and experimental research could usefully investigate the relationship between social comparisons and social media use. Participants for this study were recruited via a web-based platform from a community sample; therefore, information on objective diagnoses and severity was not obtainable, and this prevented the investigation of treatment factors and clinical severity. It is possible that some participants did not have acne and may have had other undiagnosed skin conditions. Also, there were a greater proportion of females in the Instagram group and a higher proportion of people 25 years or older (considered to have adult acne [49]) in the Facebook group, which may have affected the results. However, the majority of individuals with acne tend to self-manage [51], and consequently, the use of a community sample has a number of merits in reaching a wider range of people living with the condition. A final important limitation is that social media use was self-reported, which may have affected the reliability of the data obtained. This could be addressed in future experimental studies.

Nevertheless, the finding that appearance comparisons were associated with stigmatization in both Facebook and Instagram users and mediated the relationship between relative Facebook photo activity and stigmatization provides further support for the important role of skin-specific appearance comparisons in the psychosocial well-being of individuals living with acne, as reported in qualitative research [5,52]. Therefore, the role of upward appearance comparisons on feelings of stigmatization should be considered when working with individuals with acne-related distress. Future research could explore whether this relationship is present in populations with other skin conditions and further explore the relationship with other measures of psychological distress, such as shame, depression, and social anxiety. Given that self-compassion was consistently related to lower levels of stigmatization, interventions based on increasing self-compassion provide an additional avenue for exploring ways of reducing feelings of stigmatization in individuals with acne.

## Abbreviations

DACS: Downward Appearance Comparison Scale
UPACS: Upward Appearance Comparison Scale
